# Development and Validation of a Contrast-Enhanced CT-Based Radiomics Nomogram for Prediction of Therapeutic Efficacy of Anti-PD-1 Antibodies in Advanced HCC Patients

**DOI:** 10.3389/fimmu.2020.613946

**Published:** 2021-01-08

**Authors:** Guosheng Yuan, Yangda Song, Qi Li, Xiaoyun Hu, Mengya Zang, Wencong Dai, Xiao Cheng, Wei Huang, Wenxuan Yu, Mian Chen, Yabing Guo, Qifan Zhang, Jinzhang Chen

**Affiliations:** ^1^ Department of Infectious Diseases and Hepatology Unit, Nanfang Hospital, Southern Medical University, Guangzhou, China; ^2^ Department of Oncology, Nanfang Hospital, Southern Medical University, Guangzhou, China; ^3^ Department of Oncology, ShunDe Hospital, Southern Medical University, Guangzhou, China; ^4^ Department of Transplant Immunology Laboratory, Churchill Hospital, Oxford University Hospitals NHS Foundation Trust, Oxford, United Kingdom; ^5^ Department of Hepatobiliary Surgery, Nanfang Hospital, Southern Medical University, Guangzhou, China

**Keywords:** hepatocellular carcinoma, programmed death receptor-1, computed tomography, radiomics, nomogram

## Abstract

**Background:**

There is no study accessible now assessing the prognostic aspect of radiomics for anti-PD-1 therapy for patients with HCC.

**Aim:**

The aim of this study was to develop and validate a radiomics nomogram by incorporating the pretreatment contrast-enhanced Computed tomography (CT) images and clinical risk factors to estimate the anti-PD-1 treatment efficacy in Hepatocellular Carcinoma (HCC) patients.

**Methods:**

A total of 58 patients with advanced HCC who were refractory to the standard first-line of therapy, and received PD-1 inhibitor treatment with Toripalimab, Camrelizumab, or Sintilimab from 1st January 2019 to 31 July 2020 were enrolled and divided into two sets randomly: training set (n = 40) and validation set (n = 18). Radiomics features were extracted from non-enhanced and contrast-enhanced CT scans and selected by using the least absolute shrinkage and selection operator (LASSO) method. Finally, a radiomics nomogram was developed based on by univariate and multivariate logistic regression analysis. The performance of the nomogram was evaluated by discrimination, calibration, and clinical utility.

**Results:**

Eight radiomics features from the whole tumor and peritumoral regions were selected and comprised of the Fusion Radiomics score. Together with two clinical factors (tumor embolus and ALBI grade), a radiomics nomogram was developed with an area under the curve (AUC) of 0.894 (95% CI, 0.797–0.991) and 0.883 (95% CI, 0.716–0.998) in the training and validation cohort, respectively. The calibration curve and decision curve analysis (DCA) confirmed that nomogram had good consistency and clinical usefulness.

**Conclusions:**

This study has developed and validated a radiomics nomogram by incorporating the pretreatment CECT images and clinical factors to predict the anti-PD-1 treatment efficacy in patients with advanced HCC.

## Highlights

-Question: Is radiomics nomogram extracted from contrast-enhanced CT useful in predicting the anti-PD-1 treatment efficacy in patients with advanced HCC?
-Pertinent Findings: The nomogram, including embolus, ALBI grade and fusion radiomics score based on both tumor and peritumoral area, achieved the best performance in predicting the probability of PD after PD-1 inhibitor therapy.
-Implications for Patient Care: Our study has developed and validated a radiomics nomogram by incorporating the pretreatment CECT images and clinical factors to predict the anti-PD-1 treatment efficacy in patients with advanced HCC.

## Introduction

Hepatocellular carcinoma (HCC) is now the fourth most common cancer and the second leading causes of cancer-related mortality worldwide (1, 2). Late diagnosis, limited treatment options and lack of predictors of anti-tumor efficacy greatly account for the poor prognosis of HCC (3–5). Recently, immune checkpoint blocker therapy, particularly antibodies targeting the programmed cell death-1 (PD-1)/programmed cell death ligand-1 (PD-L1) pathway, has sparked a boom in systemic treatment aimed at improving the tumor response and survival of HCC patients (6–12). However, only a fraction of patients benefits from the PD-1/PD-L1 monotherapy, indicating it is very important to excavate a curative effect predictor for precise immunotherapy of advanced HCC (13–17). According to the limited researches (18–21), tumor mutational burden (TMB) and PD-L1 expression are the most extensively studied predictive markers for the efficacy of PD-1/PD-L1 treatment. Nevertheless, the percentage of patients with high TMB was low in HCC, and its value as a predictive marker for PD-1 therapy is not reported in the CheckMate-040 and KEYNOTE-224 study (13, 14). On the other hand, many studies have demonstrated that PD-L1 expression is associated with poor prognosis in HCC individual, while its positive expression (with a cut-off of ≥1%) occurred only in about 20% of HCC patients (13, 14, 18, 22). Besides, good anti-tumor response is usually observed during the clinical application of anti-PD-1 monotherapy even in patients with negative PD-L1 expression (23, 24). Therefore, identifying robust predictors as useful tools to predict response to PD-1/PD-L1 treatment in HCC patients is urgently needed in the era of precision medicine.

In a developing country like China, computed tomography (CT) is widely applied as an indispensable tool for differential diagnosis, treatment option determination, and therapeutic evaluation, other than magnetic resonance imaging (MRI). In China, the waiting time for CT examination is 1–2 days even in tertiary general hospitals, but when it comes to MRI examination, it can be 3–7 days at least. Moreover, radiomics has recently been recognized as a newly emerging form of imaging technology in oncology using a series of statistical analysis tools or data-mining algorithms on high-throughput imaging features to obtain predictive or prognostic information (25). Its application has achieved successful prediction abilities in various tumors by building appropriate models with refined features and clinical data (26–30). For instance, radiomic features extracted from contrast-enhanced CT (CECT) have been proved to be useful in predicting microvascular invasion (MVI) and the long-term clinical outcomes in patients with HCC (31). However, to the best of our knowledge, there is no study accessible now assessing the prognostic aspect of radiomics for anti-PD-1 therapy for patients with HCC.

The aim of our current study was to develop and validate a radiomics nomogram by incorporating the pretreatment CECT images and clinical risk factors to estimate the anti-PD-1 treatment efficacy in patients with advanced HCC.

## Material and Methods

### Study Design and Participants

This study included patients with advanced HCC who were refractory to the standard first-line of therapy and received PD-1 inhibitor treatment with Toripalimab, Camrelizumab, or Sintilimab from 1st January 2019 to 31 July 2020 in the department of Infectious diseases and Hepatology Unit, Nanfang hospital, Southern Medical University. The inclusion criteria were listed as follows: 1. patients who were aged ≥18 with HCC diagnosed by two imaging modalities, or biopsy; 2. were refractory to the standard first-line of therapy; 3. were in stage C according to the Barcelona Clinic Liver Cancer (BCLC) staging system; or in stage B who could not tolerate further surgery or ablation; 4. with Child-Pugh A or B liver function; 5. Eastern Cooperative Oncology Group (ECOG) performance status of ≤3; 6. ≥1 measurable disease at baseline per modified response evaluation criteria in solid tumor (mRECIST); 7. without heart, lung or kidney dysfunction, and life expectancy of ≥3 months.

**Figure 1:**
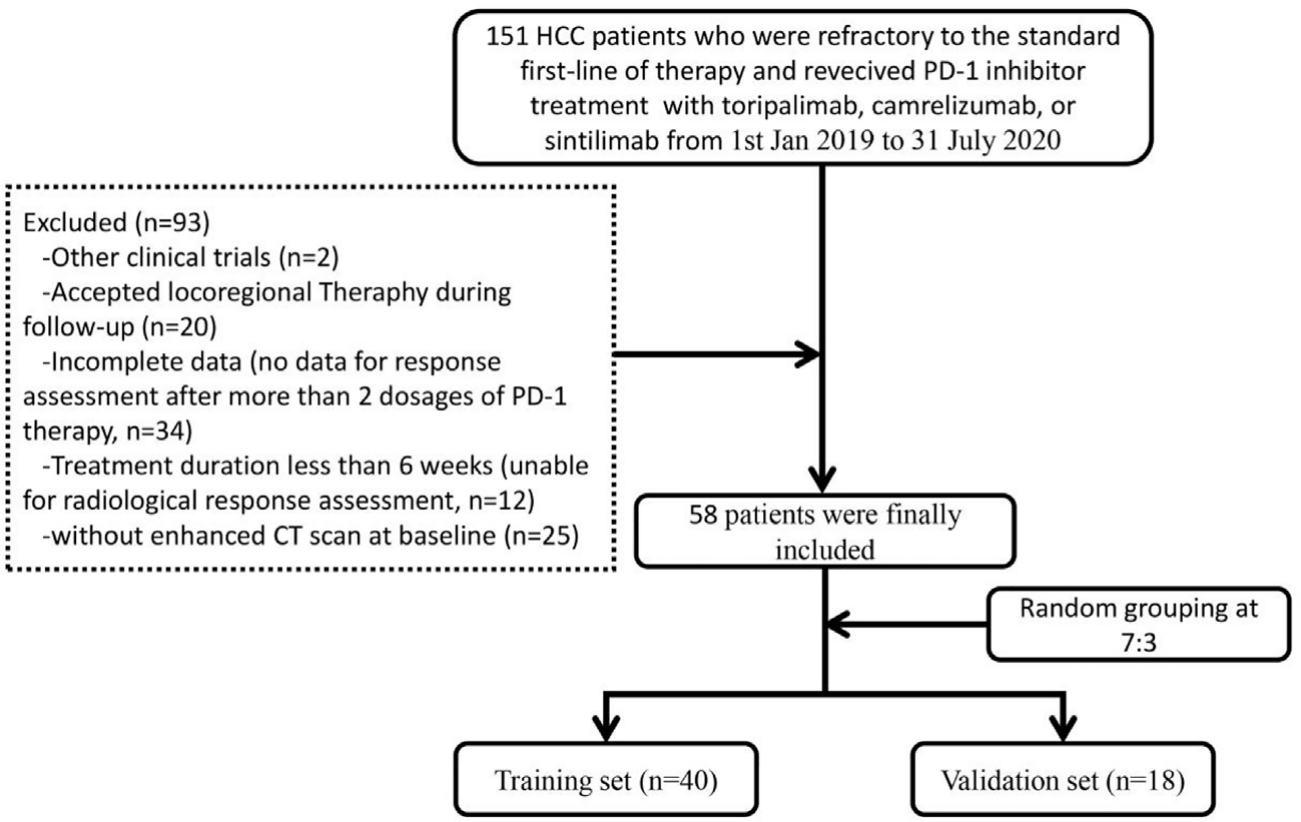
Flowchart of the controlled study patients.

Patients with the following characteristics were excluded: 1. without enhanced CT scan at baseline; 2. accepted locoregional therapy during follow-up; 3. brain or leptomeningeal metastasis or uncontrolled medical disorders that could jeopardize the outcomes of the study; 4. women who were pregnant or breast feeding; 5. currently had or had a history of malignant tumors in addition to HCC; 6. positive HAV/HCV/HDV/HIV serology; 7. attended other clinical trials. Finally, 58 HCC patients with complete data were included and divided into two sets randomly (at a ratio of 7:3): training set (n = 40) and validation set (n = 18). The reliability of this study was evaluated by calculating a power of the test based on sample sizes and the research outcomes in the two sets (32). **Figure 1** illustrates the flowchart of the enrolled study patients.

This retrospective study was approved by the ethical committee of Nanfang Hospital, Southern Medical University (NFEC-201903-Y1-01). Written consent for PD-1 inhibitor therapy was obtained from patients prior to their enrolment into this study.

### Dosage of PD-1 Inhibitor Therapy

Toripalimab was given intravenously at 3 mg/kg body weight or at a fixed dose of 240 mg every 2 weeks. Camrelizumab was given at a fixed dose of 200 mg every 2–3 weeks intravenously. Sintilimab was given at a fixed dose of 200 mg every 3 weeks intravenously.

### Clinical Data and Assessments

Clinical and laboratory data were collected from all patients prior to PD-1 inhibitor therapy. Clinical data included age, gender, Barcelona Clinic Liver Cancer (BCLC) stage, Eastern Cooperative Oncology Group (ECOG) performance, and Child-Pugh score. In addition, imaging data were collected based on abdominal computed tomography (CT) and/or magnetic resonance imaging (MRI), and included tumor size, number, vascular invasion, and extrahepatic metastasis. Laboratory data included alpha-fetoprotein (AFP), alanine aminotransferase (ALT), aspartate aminotransferase (AST), albumin, total bilirubin (TBIL), and prothrombin time (PT). Albumin-bilirubin (ALBI) score was calculated for each patient by the following formula: ALBI score = (log_10_ bilirubin × 0.66) + (albumin × -0.085), where bilirubin is in µmol/L and albumin in g/L (33).

The patients underwent CT at baseline and 2.8 (1.2, 6.2) months thereafter. TRAEs were recorded at every visit according to the US National Cancer Institute (NCI) Common Terminology Criteria for Adverse Events (CTCAE v. 4.03). The CT acquisition parameters are presented in the **Supplementary Materials**. Tumor responses were evaluated according to the modified response evaluation criteria in solid tumor (mRECIST) (34) and included the following classifications: (I) complete response (CR), disappearance of target lesions according to all enhanced imaging in the arterial phase; (II) partial response (PR), the total reduction of the diameter of the target lesions (enhanced arterial phase) by ≥30%; (III) stable disease (SD), the diameter of the target lesion not reduced to that in PR and not increased to that in progressive disease (PD); (IV) PD, the diameter of the target lesion increased by at least 20% compared with the baseline value or the appearance of new lesions according to enhanced imaging in the arterial phase.

### Image Segmentation and Radiomics Feature Extraction

**Figure 2:**
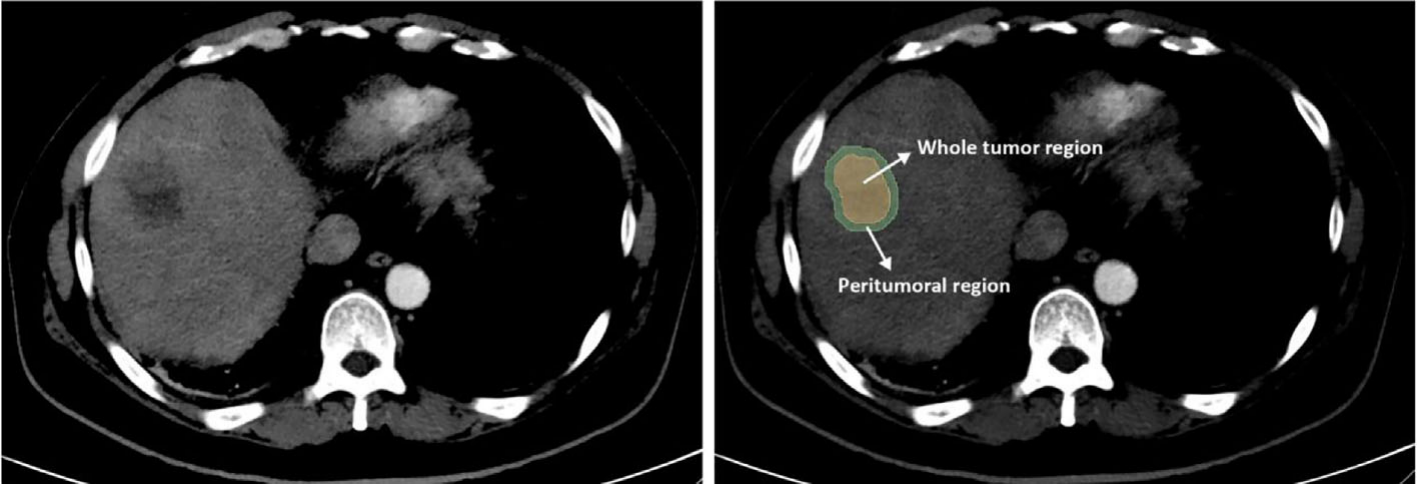
Image segmentation of radiomics. The whole tumor region (WTR) was manually segmented along the contour of the tumor on the axial slice of plain and contrast-enhanced CT. Then, a region with 10 mm distance to the tumor contour was automatically created by the 3D Slicer software and defined as the peritumoral region (PTR).

Non-enhanced and arterial phase CT images at 1.5 mm thickness were retrieved for image feature extraction. The region of Interest (ROI) including the whole tumor region (WTR) and the peritumoral region (PTR). The tumor area was manually segmented along with the contour of the tumor on the axial slice of non-enhanced and contrast-enhanced CT by two radiologists (reader 1 and reader 2, both with more than ten years of experience) who were blinded to the clinical outcome using 3D Slicer (version 4.10.2, https://www.slicer.org/) independently. Then, an automated software generated a circumferential region of interest 10mm beyond the measured tumor contour (**Figure 2**). Considering that the patients treated with PD-1 inhibitor were mostly in advanced stages with an average tumor size of 8.38 ± 3.95 cm and 9.29 ± 4.08 cm in the training and validation set respectively, we chose 10 mm as the border to avoid the inclusion of too many parts out of liver without adding any useful information. Both reader 1 and reader 2 repeated the same procedure two weeks later to evaluate the intra-observer reproducibility. And inter-observer reproducibility was evaluated between the two readers.

All voxel sizes of images were resampled with the same size of 1×1×1 mm^3^ and the grey level was normalized to a scale of 1 to 32. Wavelet filtering were applied to all the images. Then, a total of 3160 radiomics features were extracted from the two ROIs of the non-enhanced and contrast-enhanced CT by Pyradiomics (version 3.0, an open-source python package) (35). These features included 7 types: 1) Shape; 2) First order statistics; 3) Gray Level Cooccurrence Matrix; 4) Gray Level Run Length Matrix; 5) Gray Level Size Zone Matrix; 6) Neighboring Gray Tone Difference Matrix; 7) Gray Level Dependence Matrix.

### Feature Selection and Radiomics Score Calculation

Before further analysis, all the extracted radiomics features were standardized into a normal distribution with z-scores to eliminate the differences in the value scales of the data. The training set was used to build a radiomics model as the therapeutic effect of PD-1 inhibitor classifier. To avoid overfitting, feature selection is essential since the relatively low-dimensional sample size contrasted to the high-dimensional radiomics features. Firstly, we calculated the intraclass correlation coefficients (ICCs) between the features extracted from the two radiologists and features with either intra-observer or inter-observer ICCs less than 0.75 were excluded due to the relatively low robust. Secondly, we performed Spearman’s correlation test and features with the coefficients greater than 0.95 were excluded due to the redundancy. Thirdly, among the remaining features, features with significant differences between PD and non-PD (CR + PR + SD) groups were selected through *t* test. Fourthly, we applied the least absolute shrinkage and selection operator (LASSO) method to select the most powerful features in the training set. Finally, a radiomics score (Fusion Rad-score) based on combination ROIs of the tumor and peritumoral area (WTR+PTR) was calculated for each patient based on a liner combination of the selected radiomics features weighted by their LASSO coefficients. Similarly, a radiomics score (Tumor Rad-score) based only on the ROI of the tumor area (WTR) was also calculated. In addition, we also tried several different machine learning classifiers including the LASSO, Random forest, Support vector machines and Decision tree. It turns out that LASSO had the best AUC and F1-score. Details of the results had been added to the **Supplementary Materials**.

### Development and Validation of Combination Nomogram

Clinical characteristics and radiomics score were selected through univariate and multivariate logistic regression analysis and a nomogram was built based on the independent risk factors in the multivariate analysis. Similarly, a combined model (called combined model 2) was built based on independent clinical factors and Tumor Rad-score. Multi-collinearity was evaluated by the variance inflation factors (VIFs) for variables in the nomogram and the combined model 2. Variables with VIFs > 10 indicated severity multicollinearities (36). The discrimination performance of the nomogram and combined model 2 was evaluated by Harrell’s c-index and receiver operator characteristic (ROC) curves in the training and validation sets (37). Comparison between different ROC curves was performed by Delong test (38). The predictive accuracy of the two models was evaluated by calibration curves and the Hosmer-Lemeshow test (39). Decision curve analysis (DCA) was performed to determine the clinical utility of two models and a larger area under the curve indicated a better clinical utility (40). Additionally, a self-evaluated radiomics quality score is presented in the **Supplementary Materials**.

### Statistical Analysis

Statistical analyses were performed by SPSS (version 26, Chicago, IL, USA) and R software (version 3.6.2, http://www.Rproject.org). In the comparison of baseline data, we used the Student *t* test or Mann-Whitney *U* test for continuous variables, and the *χ*2 test or Fisher’s exact test for categorical variables. Correlation analysis was assessed by the Spearman correlation test. Nomogram and calibration curves were plotted by using the “RMS” package. The ROC curves were plotted by using the “pROC” package and the DCA curves were plotted by using the “RMDA” package. For all tests, two-sided *P* < 0.05 was considered statistically significant.

## Results

### Baseline Characteristics of the Training and Validation Set

A total of 58 patients received 3 different PD-1 inhibitor treatment based on the antibody used: toripalimab (n = 20), camrelizumab (n = 27) and sintilimab (n = 11). Accordingly, 13 (32.5%), 19 (47.5%) and 8 (20.0%) patients accepted toripalimab, camrelizumab, and sintilimab, respectively, in the training set (n = 40); and 7 (38.9%), 8 (44.4%) and 3 (16.7%) respectively, in the validation set. There is no statistical difference in the proportion of patients treated with each drug in the two sets (data un-presented).

The power of our study was 0.98 and 0.70 in the training and validation set respectively, suggesting a sufficient sample size of the study and a credible conclusion. The baseline patient characteristics of the training cohort and the validation sets are shown in **Table 1**. There were no significant differences in the baseline patient characteristics between the training set and validation set, indicating a good consistency between the two data sets. Seven factors showed significant differences between PD and non-PD in the training set: Alb, PLT, ALBI, ALBI grade, tumor embolus, Fusion Rad-score and Tumor Rad-score. Among the total 58 patients, 47 patients (81.0%) achieved tumor control (CR + PR + SD), while 11 patients (19.0%) with progressive disease. The ORR and DCR is 22.4% and 81.0%, respectively (**Table 2**)

**Table 1:** Patient characteristics in training and validation set. (n = 58).

Characteristics	Training set	Validation set	P value
Age	55.30 ± 13.28	52.00 ± 12.87	0.380
Gender			0.422
Men	35 (87.5%)	17 (94.4%)	
Women	5 (12.5%)	1 (5.6%)	
AFP (ng/ml)			0.414
<20	9 (22.5%)	5 (27.8%)	
20–400	14 (35.0%)	6 (33.3%)	
>400	17 (42.5%)	7 (38.9%)	
Alb (g/L)^Δ^	37.95 [31.73, 40.78]	39.80 [37.75, 41.00]	0.377
PLT (10^9^/L)	203.97 (103.86)	166.33 (56.18)	0.155
Tbil (µmol/L) ^Δ^	16.30 [12.62, 25.40]	13.85 [12.05, 18.12]	0.378
Child-pugh grade			0.400
A	15 (68.2%)	12 (66.7%)	
B	4 (18.2%)	4 (22.2%)	
C	3 (13.6%)	2 (11.1%)	
ALBI^Δ^	-2.48 [-2.70, -1.94]	-2.63 [-2.75, -2.30]	0.201
ALBI grade			0.5
1	16 (40.0%)	10 (55.6%)	
2	16 (40.0%)	6 (33.3%)	
3	8 (20.0%)	2 (11.1%)	
Tumor number			1.000
<3 nodules	9 (22.5%)	4 (22.2%)	
≥3 nodules	31 (77.5%)	14 (77.8%)	
Tumor size (cm)	8.38 ± 3.95	9.29 ± 4.08	0.423
Embolus			0.585
Absent	18 (45.0%)	6 (33.3%)	
Present	22 (55.0%)	12 (66.7%)	
Fusion Rad-score	-0.07 ± 1.73	-0.01 ± 1.80	0.903
Tumor Rad-score	0.01 ± 2.23	-0.16 ± 1.72	0.770
Tumor responses			0.948
CR	1 (2.5%)	0 (0.0%)	
PR	8 (20.0%)	4 (22.2%)	
SD	24 (60.0%)	10 (55.6%)	
PD	7 (17.5%)	4 (22.2%)	

^Δ^For abnormal distribution, data are shown as medians [interquartile ranges] and were analyzed by the Kruskal-Wallis H test.

ALBI, albumin-bilirubin; ALBI score = (log_10_ bilirubin × 0.66) + (albumin × -0.085); CR, complete response; PR, partial response; SD, stable disease; PD, progressive disease.

**Table 2:** Tumor Responses for all PD-1 treated patients.

Tumor response	All patients (n = 58)
Complete response (CR)	1
Partial response (PR)	12
Stable disease (SD)	34
Progressive disease (PD)	11 (19.0%)
ORR (CR + PR)	22.4%
DCR (CR + PR + SD)	81.0%

CR, Complete response; PR, Partial response; SD, Stable disease; PD, Progressive disease; ORR, Objective response rate; DCR, Disease control rate.

### Feature Selection

**Figure 3:**
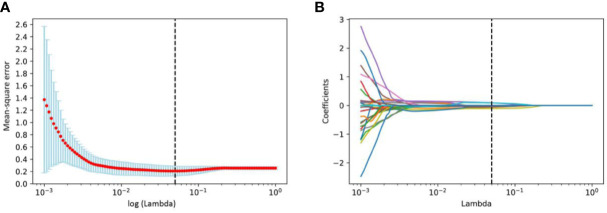
Radiomics features selection using the least absolute shrinkage and selection operator (LASSO) binary logistic regression model. **(A)** Tuning parameter log (λ) selection in the LASSO model used 10-fold cross-validation via minimum criteria. And the vertical black dashed line represents the lowest mean-square error corresponds to log (λ) is 0.0498. **(B)** LASSO coefficient profiles of all the radiomics features. Vertical black dashed line represents the optimal λ resulted in nine nonzero features.

**Figure 4:**
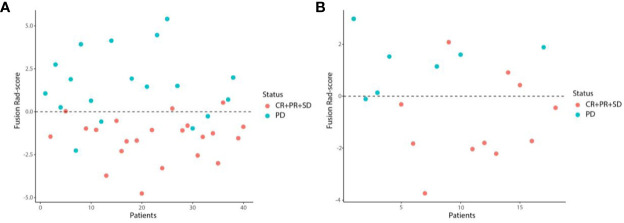
Scatter plots of the Fusion Rad-score in the training **(A)** and validation **(B)** set. Patients with Fusion Rad-score higher than 0 are classified as PD.

We first excluded features with ICCs less than 0.75, and features of WTR and PTR was reduced to 1432 and 1360 respectively. Among the remaining features, 732 and 650 features of WTR and PTR were retained with correlation coefficients greater than 0.9 by Spearman’s correlation test. Then, 26 and 17 features of WTR and PTR showed significant differences between PD and non-PD (CR + PR + SD) groups *via t* test in the training set. Finally, 6 and 3 features were selected *via* LASSO regression (**Figure 3**). Fusion Rad-score (**Figure 4**) and Tumor Rad-score were calculated and the detailed formulas was provided in **Supplementary Material 1**. Compared to patients with non-PD, patients with PD after PD-1 inhibitor treatment had higher Fusion Rad-score (0.8 ± 1.6 vs. -0.8 ± 1.6, *P* = 0.002) in the training set. The inter-observer reproducibility was high and more details of the intra- and inter-observer reproducibility are shown in **Supplementary Material 3**.

### Identification of Independent Risk Factors for PD After PD-1 Inhibitor Treatment

**Figure 5:**
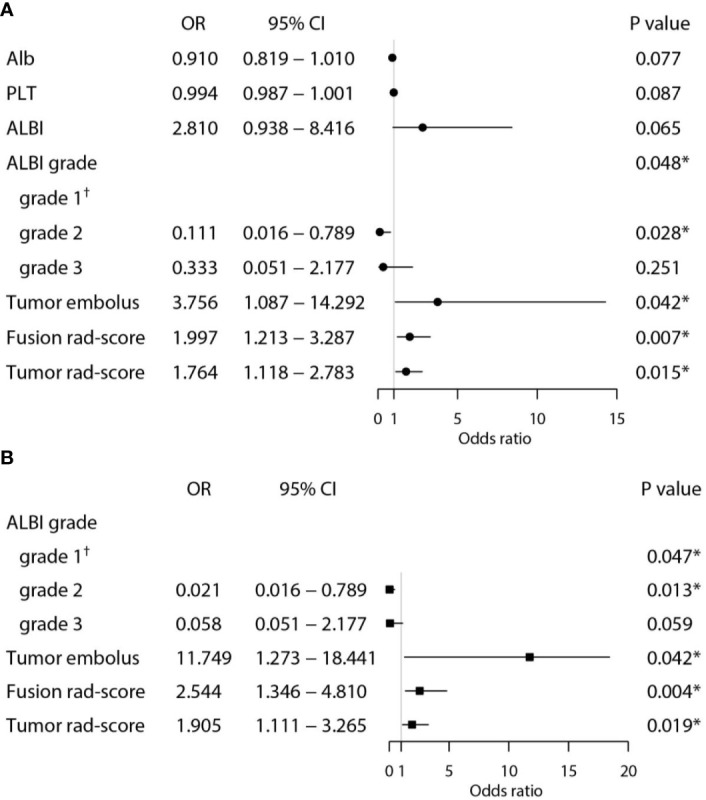
Univariate **(A)** and multivariate **(B)** analyses for predicting PD in the training set. (*Statistically significant results from logistic regression analysis; ^†^Used as the reference category).

Univariate and multivariate logistic regression analysis showed that tumor embolus, ALBI grade, Fusion rad-score, and Tumor rad-score were found to be independent risk factors for PD after PD-1 inhibitor treatment (**Figure 5**). The VIFs in the nomogram (1.008, 1.006, and 1.010) and combined model 2 (1.053, 1.042, and 1.088) were all less than 10, indicating there was no multicollinearity among these variables.

### Development and Validation of the Nomogram

**Figure 6:**
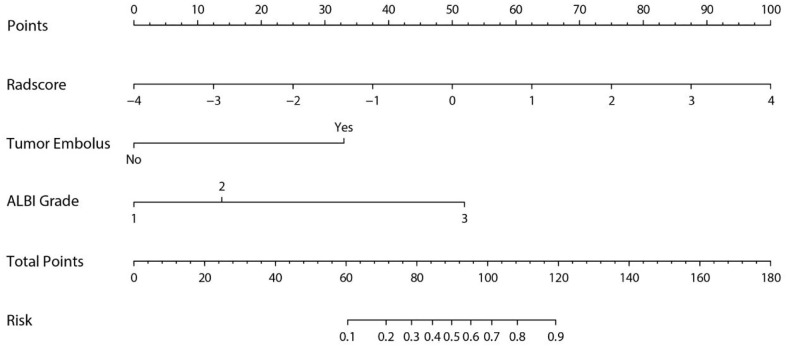
Nomogram for predicting probability of PD after PD-1 inhibitor therapy of HCC.

**Figure 7:**
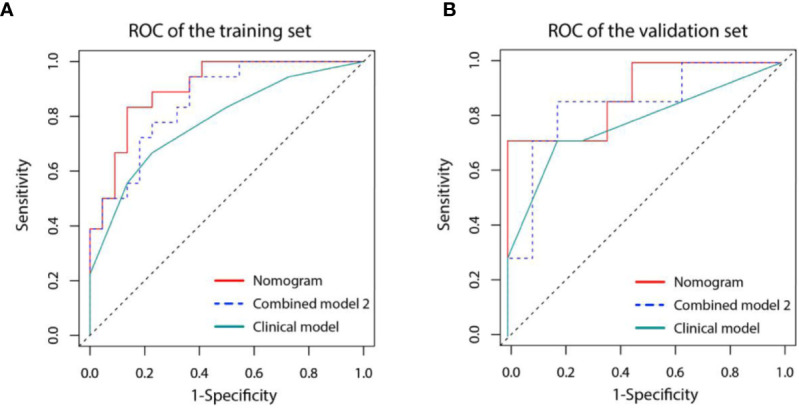
Receiver operating characteristic (ROC) curves for three models in the training **(A)** and validation **(B)** set. Model based on only clinical factors (green line) includes embolus and ALBI grade. Combined model 2 (blue line) contains the above two clinical factors and Tumor Radiomics score based on only tumor area. Nomogram (red line) contains clinical factors and Fusion Radiomics score based on both tumor and peritumoral area.

**Figure 8:**
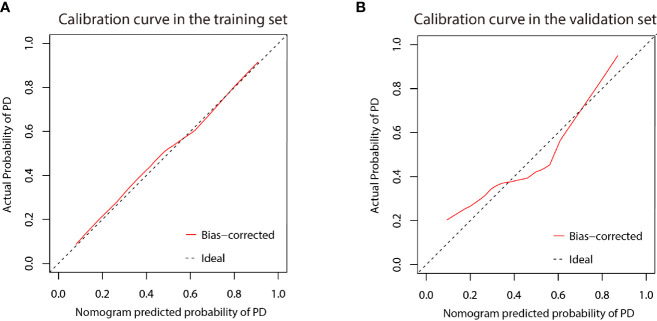
Calibration curve of the nomogram in the training **(A)** and validation **(B)** cohort. X-axis represents the nomogram predicted probability of PD. Y-axis represents the actual probability of PD, and the diagonal dashed line (represent ideal) indicates the ideal prediction by a perfect model. Results were plotted via bootstrapping with 1000 resamples. The closer the bias-corrected calibration curve (red line) is to the diagonal line, the higher the prediction accuracy of the model.

**Figure 9:**
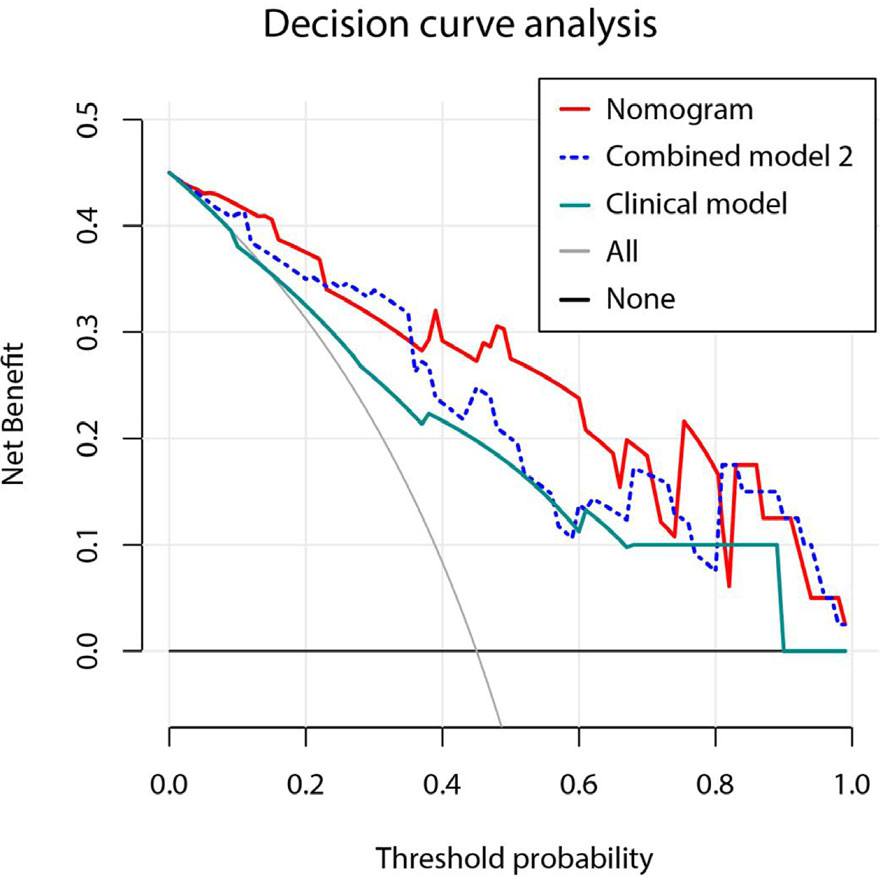
Decision curve analysis for three models. Model based on only clinical factors includes embolus and ALBI grade. Combined model 2 contains the above two clinical factors and Tumor Radiomics score based on only tumor area. Nomogram contains clinical factors and Fusion Radiomics score based on both tumor and peritumoral area. Result shows that using the nomogram for PD prediction has more benefit than two extreme condition (the treat-all-patients scheme (gray curve) and the treat-none scheme (horizontal black line)). A larger area under the decision curve suggested a better clinical utility. Nomogram (red line) received a higher net benefit than combined model 2 (blue dashed line) and the model based on clinical factors (green line).

A nomogram was established based on the results of multivariate logistic regression (**Figure 6**). Variables of the nomogram included two clinical factors (tumor embolus and ALBI grade) and Fusion Rad-score. Harrell’s C-index was 0.851 and 0.791 respectively (*P*>0.05) in the training and validation set. In the training set, the nomogram yielded an AUC of 0.894 (95% CI, 0.797–0.991) with a sensitivity of 83.3% and a specificity of 86.4%. In the validation set, the nomogram exhibited an AUC of 0.883 (95% CI, 0.716–0.998) with a sensitivity of 71.4% and a specificity of 81.8% (**Figure 7**). The nomogram showed better performance than combined model 2 or clinical model in the training set (AUC: 0.894 vs. 0.846 or 0.740, P = 0.1401 and P = 0.037) and validation set (AUC: 0.883 vs. 0.831 or 0.739, P = 0.563 and P = 0.044). Calibration curves (**Figure 8**) and Hosmer-Lemeshow test indicated good consistency between the nomogram-predicted probability of PD and the actual PD rate in both sets (P = 0.758 and P = 0.537). DCA demonstrated a higher net benefit of the nomogram than combined model 2 and the model based on clinical factors, indicating that treatment strategies based on our nomogram prediction have a better clinical utility (**Figure 9**).

## Discussion

In the present study, we have successfully developed and validated a radiomics nomogram for the pretreatment individualized prediction of anti-tumor efficacy in patients with advanced HCC and received PD-1 inhibitor therapy with three domestic drugs: Toripalimab, Camrelizumab, and Sintilimab. The nomogram, including embolus, ALBI grade and fusion radiomics score based on both tumor and peritumoral area, achieved the best performance in predicting the probability of PD after PD-1 inhibitor therapy.

According to the results of two pioneering PD-1 applications in HCC patients, nivolumab and pembrolizumab both showed an unsatisfactory DCR of 64% and 62%, respectively (13, 14), meaning that nearly 30–40% patients failed to respond to anti-tumor therapy (also means progressive disease, PD). Unfortunately, there are currently no reliable predictive biomarker to aid in the precision of PD-1/PD-L1 therapy and excavating a curative effect predictor as useful tool for PD-1/PD-L1 application in HCC patients is urgently needed in the era of precision medicine. Higher PD-L1 expression of tumor cells have been showed to be associated with a better objective response to pembrolizumab, while good anti-tumor response to nivolumab occurred in patients regardless of PD-L1 expression (18, 19). Additionally, patients with high level of TMB prior to starting therapy may respond to immune checkpoint inhibition (20). Nevertheless, the percentage of patients with high TMB was low in HCC, and its application as a predictive marker for PD-1 therapy is not recommended in our current clinical practice (20, 21). Therefore, more studies are needed to identify robust predictors as useful tools to allow clinicians to tailor therapy for patients who may fail to respond to PD-1/PDL1 inhibition.

In recent years, advances in machine learning have changed the traditional way of cancer research and thinking. In the era of numerous data, how to use these data effectively, especially information that cannot be directly recognized by the human brain, is the hotspot of research. Radiomics was first proposed by Lambin in 2012 (41), which enables quantification of diseases by extracting massive features from images (including CT, MRI, PET-CT, and so on) and ultimately assists the physician in making the most accurate diagnosis. Since then, more and more studies have applied it in the field of cancer research, especially in diagnosis and efficacy prediction. Zhang Z et al. established a radiomics nomogram based on gadoxetic acid-enhanced MRI showed an AUC of 0.844 in the preoperative prediction of early HCC recurrence after surgery (42). In another study, based on another way of model construction, Ji GW et al. found a radiomics approach demonstrated a better performance in predicting lymph node metastasis in intrahepatic cholangiocarcinoma, especially in CT-reported LN-negative subgroup with an AUC of 0.922 (43). In the field of HCC, most research relative with radiomics are applied for predicting outcome or treatment response after surgery (44–46). PD-1 immunotherapy is being used more and more widely in patients with advanced liver cancer who have lost the opportunity for surgery. In our study, we develop a radiomics nomogram constructed by incorporating Fusion Rad-score from radiomics method and two clinical features including ALBI grade and tumor embolus to predict tumor response after PD-1 inhibitor treatment. Comparing with the existing radiomics nomogram in HCC, our current study has several benefits: 1. To our knowledge, this is the first tool to predict tumor response after PD-1 inhibitor treatment in HCC patients. 2. It provided user-friendly scoring system which could help physicians to select patients who might benefit from PD-1 inhibitor treatment. 3. The tumor area and peritumoral area were both selected to cover the heterogeneous cells and their surrounding microenvironment in our study. As previous imaging studies were mostly based on the shape, density and enhancement of tumors, which did not quantify the information of the images and were easily affected by the subjectivity of the radiologists.

In the variables included by the nomogram, tumor thrombus has been widely recognized as a significant poor prognostic factor for HCC (47). According to the BCLC Staging system, HCC patients with portal vein tumor thrombus are classified as stage C and sorafenib is the only recommended treatment. Tumor thrombus may be seen as areas of solid or streaky arterial phase enhancement within the portal vein and its branches after contrast administration on CT. As another independent risk factor, the ALBI grade, which based Alb and Tbil levels, has been widely recognized as a good indicator for assessing liver function and predicting the prognosis of HCC (33). Ding M et al. found that ALBI grade is an independent risk factor for overall survival in patients with HCC after thermal ablation (48). Fan R et al. built a prediction model which incorporates ALBI score to predicts HCC development in patients with chronic hepatitis (49). This model was developed based on more than 17,000 patients from 11 global prospective studies and yielded a C-index of 0.82–0.87.

Many of the recent radiomics studies only focus on the feature extraction of primary foci and ignore the peritumor microenvironment. In our study, we both extracted features from the tumor area and peritumoral area. And the Fusion Rad-score which consisted features from the peritumoral area exhibited better performance than the Tumor Rad-score. In line with the study by Rui Z et al. (50), this result indicated that the combination of peritumoral features provide more information of the tumor microenvironment, which can reflect the biological behavior of the tumor better.

It is worth noting that 88.9% (eight of nine) of the features consisted of the Fusion Rad-score were wavelet features. The wavelet transformation is a mathematical technique which can decompose special patterns hidden in mass of data. Our finding was consistent with previous studies, which indicated the important role of wavelet transformation in mining the hidden patterns from various data (51). A recent radiomics study employed the support vector machine methods to predict preoperative lymph node status in intrahepatic cholangiocarcinoma, and the five image features selected were all wavelet features (52). With the wavelet transformation, it makes quantification of intratumoral heterogeneity at different scales become possible, which are often invisible to the naked eye.

There are several limitations to our study. First, another important imaging modality, MRI, was not included in the current study according to the shortages we had mentioned in the Introduction section of this paper. Further studies are needed to determine whether the developed model is suitable for MRI in PD-1/PD-L1 treatment efficacy prediction. Second, this study was retrospectively designed, although objective endpoints (especially imaging data for tumor responses assessment) were carefully and integrally recorded. Third, the sample size was relatively small and the survival data was not included in the present study, as the three domestic anti-PD-1 antibodies have only been applied in our clinical practice for just over a year. Our future study will expand the sample size and focus on the subgroup analysis of survival.

The findings in our study are important as we firstly demonstrate that radiomics signatures of CECT in cooperation with clinical characteristics are useful in predicting the efficacy of the three domestically developed PD-1 antibodies in treating Chinese HCC patients. However, these data should not be taken as non-biased or used to inform clinical decision-making without further evidence-based confirmation. Therefore, better controlled, prospective and larger sample size cohorts to assess the clinical utility of this new radiomic tool for HCC patients would be needed. In conclusion, this study has developed and validated a radiomics nomogram by incorporating the pretreatment CECT images and clinical factors to predict the anti-PD-1 treatment efficacy in patients with advanced HCC.

## Data Availability Statement

The raw data supporting the conclusions of this article will be made available by the authors, without undue reservation.

## Ethics Statement

The studies involving human participants were reviewed and approved by Nanfang Hospital, Southern Medical University. The patients/participants provided their written informed consent to participate in this study.

## Author Contributions

(I) Conception and design: GY, YS, JC. (II) Administrative support: JC, YG. (III) Provision of study materials or patients: XH, QL, WD, XC, YG, JC, WH, WY. (IV) Collection and assembly of data: GY, YS. (V) Data analysis and interpretation: GY, YS, YG, XH, MC, JC. (VI) Manuscript writing: All authors. (VII) Manuscript revised: GY, YS, JC, and QZ. All authors contributed to the article and approved the submitted version.

## Funding

This study was partly supported by the grants from Natural Science Foundation of Guangdong Province (2017A030313645), Self Financing Science and Technology Project of Foshan City (2018AB00963) and WBE Liver Fibrosis Foundation (CFHPC2020031). The funding agencies had no role in study design, data collection and analysis, decision to publish, or preparation of the manuscript.

## Conflict of Interest

The authors declare that the research was conducted in the absence of any commercial or financial relationships that could be construed as a potential conflict of interest.
