# Supplementary material for: Development and Validation of a Contrast-Enhanced CT-Based Radiomics Nomogram for Prediction of Therapeutic Efficacy of Anti-PD-1 Antibodies in Advanced HCC Patients
Source: Front Immunol. 2021 Jan 8;11:613946. doi: 10.3389/fimmu.2020.613946 (PMC7820863; doi:10.3389/fimmu.2020.613946)
Supplement: Supplementary file 1 [file DataSheet_1.docx]

**Supplementary Material 1.** CT acquisition parameters:

All CT images were acquired using a 64-multidetector CT imaging system (SOMATOM Definition, SIEMENS). The detailed parameters are as follows: 1) tube current:130-280 mAs; 2) tube potential: 120 kV; 3) rotation time: 0.5-0.75 second; 4) pitch: 0.8; 5) beam collimation: 128*0.626 mm; 6) field-of-view: 300-400 mm 7) matrix: 512×512; 8) reconstructed slice thickness: 1 mm. For contrast–enhanced CT imaging, non-enhanced, enhanced arterial phase (25 seconds), portal venous phase (70 seconds) and delayed phase (3 minutes) were obtained. All patients received an injection of ionic contrast material (Ultravist, Bayer, Germany) at a total dose of 1.5ml/kg body weight into the cubital vein at a rate of 3.0 ml/s.

**Supplementary Material 2.** Radiomics score (Rad-score) calculation formula:

Fusion Rad-score = -0.08336659 + Non-enhanced_wavelet_LHL_firstorder_Median × -0.90497047 + Non-enhanced_wavelet_HLH_glrlm_HighGrayLevelRunEmphasis × -0.55016333 + Non-enhanced_wavelet_HLH_glrlm_LowGrayLevelRunEmphasis × 0.55016333 + Non-enhanced_p_original_shape_Elongation × -0.43820497 + Enhenced_wavelet_LLH_firstorder_Minimum × -0.37180915 + Enhenced_wavelet_HHH_glszm_SizeZoneNonUniformityNormalized × 0.79839529 + Enhenced_wavelet_LLL_glrlm_LongRunLowGrayLevelEmphasis × -0.31528712 + Enhenced_p_wavelet_LLH_firstorder_Minimum × -0.00356771 + Enhenced_p_wavelet_LLL_firstorder_Median × 0.68481702

Tumor Rad-score = -0.261036 + Non-enhanced_wavelet_LHL_firstorder_Median × -1.13739707 + Non-enhanced_wavelet_HLH_glrlm_HighGrayLevelRunEmphasis × -0.54308321 + Non-enhanced_wavelet_HLH_glrlm_LowGrayLevelRunEmphasis × 0.54308321 + Enhenced_wavelet_LLH_firstorder_Minimum × -0.55488659 + Enhenced_wavelet_HHH_glszm_SizeZoneNonUniformityNormalized × 0.70081749 + Enhenced_wavelet_LLL_glrlm_LongRunLowGrayLevelEmphasis × -0.53744504

**Supplementary Material 3. Evaluation of the intra- and inter-observer** **reproducibility**

The intra- and inter-observer reproducibility were evaluated by using the interclass correlation coefficients (ICCs). Based on the criteria of either ICCs greater than 0.75, 148 and 220 features of the whole tumor region (WTR) and the peritumoral region (PTR) were excluded (ICCs: 0.382 to 0.749; 0.401 to 0.749). Meanwhile, we evaluated the intra- and inter-observer reproducibility by using another method--Dice similarity coefficient (DSC)*. DSC is a statistical tool which measures the similarity between two sets of data [1]. Results showed that the DSCs of the intra- and inter-observer were 0.926 and 0.821 respectively, indicating good reproducibility.

Of all the features, the inter-observer reproducibility was still good with the median ICC of 0.884 (95% CI 0.809 to 0.999). Notably, the inter-observer ICCs of the nine features selected by the LASSO regression shown excellent agreement, ranging from 0.944 to 0.999.

*DSC = 2 * |X ∩ Y| / (|X| + |Y|). X and Y are two sets.[1] Anthony D. Yao , Derrick L. Cheng, Ian Pan, Felipe Kitamura; Deep Learning in Neuroradiology: A Systematic Review of Current Algorithms and Approaches for the New Wave of Imaging Technology; Radiology: Artificial Intelligence. Published Online:Mar 4 2020 doi.org/10.1148/ryai.2020190026.

Table S1. Performances of different machine learning classifiers

| Classifiers | Training set | | | | Validation set | | | |
| --- | --- | --- | --- | --- | --- | --- | --- | --- |
|  | Accuracy | Precision | AUC | F1-score | Accuracy | Precision | AUC | F1-score |
| LASSO | 73.0% | 72.3% | 0.772 | 0.723 | 69.0% | 69.6% | 0.705 | 0.690 |
| Random Forest | 64.3% | 61.2% | 0.651 | 0.614 | 57.5% | 56.6% | 0.668 | 0.485 |
| SVM | 65.9% | 65.0% | 0.711 | 0.592 | 65.0% | 66.6% | 0.691 | 0.537 |
| Decision tree | 65.7% | 57.8% | 0.655 | 0.595 | 67.5% | 65.3% | 0.678 | 0.671 |

AUC: area under the curve; SVM: Support vector machines; TP: True Positive; FP: False Positive; TN: True Negative; FN: False Negative;

Accuracy = (TP + TN) / (TP + FP + TN + FN) ;

Precision= TP / (TP + FP);

Recall=TP/(TP+FN);

F1-Score = 2 × Precision × Recall / (Precision + Recall).

F1-Score is the harmonic mean of the precision and recall. The highest possible value of an F-score is 1, indicating perfect precision and recall. ^[1]^

[2] Derczynski, L. (2016). Complementarity, F-score, and NLP Evaluation. Proceedings of the International Conference on Language Resources and Evaluation.

Table S2. Radiomics quality score according to the six key domains ^[2]^

| **Radiomics quality score** | **Score** |
| --- | --- |
| Total (36 points) | 20 |
| **Domain 1: Protocol quality and stability in image and segmentation (0 to 5 points)** | 4 |
| Protocol quality (2) | 1 |
| Test-retest (1) | 1 |
| Phantom study (1) | 1 |
| Multiple segmentation (1) | 1 |
| **Domain 2: Feature selection and validation (−8 to 8 points)** | 8 |
| Feature reduction or adjustment of multiple testing (−3 or 3) | 3 |
| Validation (−5, 2, 3, 4, or 5) | 5 |
| **Domain 3: Model performance index (0 to 5 points)** | 5 |
| Discrimination statistics (2) | 2 |
| Calibration statistics (2) | 2 |
| Cut-off analysis (1) | 1 |
| **Domain 4: Biologic/clinical validation and utility (0 to 6 points)** | 3 |
| Non-radiomics features (1) | 1 |
| Biologic correlates (1) | 1 |
| Comparison to “gold standard” (2) | 0 |
| Potential clinical utility (2) | 1 |
| **Domain 5: High level of evidence (0 to 8 points)** | 0 |
| Prospective study (7) | 0 |
| Cost-effective analysis (1) | 0 |
| **Domain 6: Open science and data (0 to 4 points)** | 0 |

[3] Lambin P, Leijenaar RTH, Deist TM et al. Radiomics: the bridge between medical imaging and personalized medicine. *Nat Rev Clin Oncol.* Dec 2017;14:749–762.
